# Novel Delivery of Mitoxantrone with Hydrophobically Modified Pullulan Nanoparticles to Inhibit Bladder Cancer Cell and the Effect of Nano-drug Size on Inhibition Efficiency

**DOI:** 10.1186/s11671-018-2769-x

**Published:** 2018-10-30

**Authors:** Xiaojun Tao, Ting Tao, Yi Wen, Jiajin Yi, Lihua He, Zixuan Huang, Yu Nie, Xiaoyan Yao, Yingying Wang, Chunlian He, Xiaoping Yang

**Affiliations:** Key Laboratory of Study and Discovery of Small Targeted Molecules of Hunan Province and Department of Pharmacy, School of Medicine, Hunan Normal University, Changsha, 410013 China

**Keywords:** Hydrophobic, Cholesterol-substituted pullulan, Nanoparticle, Size, Bladder cancer

## Abstract

Reducing the dosage of chemotherapeutic drugs via enhancing the delivery efficiency using novel nanoparticles has great potential for cancer treatment. Here, we focused on improving mitoxantrone delivery by using cholesterol-substituted pullulan polymers (CHPs) and selected a suitable nano-drug size to inhibit the growth of bladder cancer cells. We synthesized three kinds of CHPs, named CHP-1, CHP-2, CHP-3. Their chemical structures were identified by NMR, and the degree of cholesterol substitution was 6.82%, 5.78%, and 2.74%, respectively. Their diameters were 86.4, 162.30, and 222.28 nm. We tested the release rate of mitoxantrone in phosphate-buffered saline for 48 h: the release rate was 38.73%, 42.35%, and 58.89% for the three CHPs. The hydrophobic substitution degree in the polymer was associated with the self-assembly process of the nanoparticles, which affected their size and therefore drug release rate. The release of the three drug-loaded nanoparticles was significantly accelerated in acid release media. The larger the nanoparticle, the greater the drug release velocity. At 24 h, the IC_50_ value was 0.25 M, for the best inhibition of mitoxantrone on bladder cancer cells.

3-(4,5-Dimethyl-2-thiazolyl)-2,5-diphenyl-2-H-tetrazolium bromide (MTT) experiments demonstrated that drug-loaded CHP-3 nanoparticles with the largest size were the most toxic to bladder cancer cells. Immunofluorescence and flow cytometry revealed that drug-loaded CHP-3 nanoparticles with the largest size had the strongest effect on promoting apoptosis of bladder cancer cells. Also, the three drug-loaded nanoparticles could all inhibit the migration of MB49 cells, with large-size CHP-3 nanoparticles having the most powerful inhibition.

## Background

Chemotherapy is a common treatment for tumors. However, because of the lack of tissue specificity, the therapeutic effect of chemotherapy is limited and often has strong side effects [1, 2]. Therefore, research into using nanoparticle (NP) preparations to augment the target ability of chemotherapeutic drugs has increased [3–5].

After passively targeting tumor tissues via the enhanced permeability and retention (EPR) effect, nano-drugs such as NP-loaded small-molecule anti-tumor drugs mainly exert their efficacy in two ways: (1) by being released in tumor tissues and entering cells in a free form to exert efficacy and (2) by being taken up by cells in the form of microparticles and released in the cell to exert pharmacodynamic effects [6, 7]. When a nano-pharmaceutical agent is passively targeted to a tumor, which of the two methods plays a major role or whether both play a major role at the same time and whether other factors are involved is a complex issue. Because of the metabolic activity of tumor tissues, ischemia and hypoxia and the accumulation of lactic acid and because extracellular fluid of tumor tissues shows weak acidity, many nano-drugs show increased release in acidic environments, for enhanced efficacy [8]. The drug release efficiency of nano-drugs in an acidic environment is closely related to the physicochemical properties of nanomaterials and is also affected by the size of NPs [9–11]. After the NPs passively target the tumor tissue, because the tumor cells have a phagocytosis function, the nano-pharmaceutical preparation enters cells mainly via pinocytosis and complex processes mediated by the cell membrane proteins [12, 13]. Under the degradation of intracellular lysozymes, nano-pharmaceuticals release drugs and exert efficacy [14].

The uptake efficiency of target cells in targeted tissue is closely related to the properties of nanomaterials, surface modification, morphology, charge, and NP size [15–18]. Cell uptake depends to a large extent on NP size. The internalization (endocytosis) of Her-gold NPs highly depends on size, the most effective absorption occurring in NPs in the 25- to 50-nm range [19]. Extremely small or large NPs will have inefficient absorption. The 40- to 50-nm size is the critical point for receptor-mediated endocytosis [20]. In addition, NP size affects cytotoxicity. In comparing NPs of 45 and 90 nm, the size of polymer NPs is inversely related to cytotoxicity [21]. The size of NPs affects the release of the drug in tumor tissue and also the uptake efficiency of the cells and ultimately plays an important role in the efficacy of the drug.

The local adhesion of polysaccharides improves the localization and targeting function. The acidic environment of external cancer cells leads to a partial release of polysaccharide nano-drugs, triggering the dual therapeutic effect of drug-loaded NPs and free drugs after passive targeting of tumor tissues [22, 23].

Pullulan, which is non-toxic, is easily degraded in the body, and its cholesterol is an intrinsic substance in the body, so it is safe and suitable as a carrier for drugs [24, 25]. Cholesteryl hydrophobically modified pullulan (CHP) polymers, which have hydrophobic cholesteryl groups and hydrophilic sugar chains, can self-assemble into nanosphere-like structures with hydrophobic central cores and hydrophilic shells [26, 27]. Amphiphilic polymers self-assemble into NPs in spheroid structures, with the hydrophobic core formed by hydrophobic groups such as cholesteryl groups [28].

Mitoxantrone, a broad-spectrum anticancer active anthracycline antibiotic that can intercalate DNA and inhibit topoisomerase II, is a classical antitumor drug. However, because of its cardiotoxicity, mitoxantrone use is limited. Mitoxantrone is loaded onto the hydrophobic center of CHP NPs by hydrophobic interaction to form CHP nanometer preparations that have a passive targeting effect via the EPR effect. As compared with free drugs, drug-loaded CHP NPs show reduced toxic effects of drugs and increased anticancer efficiency [29, 30]. The hydrophobic group cholesteryl in the CHP polymer drives the formation of the core structure of the NP, and within a certain range, the higher the degree of substitution of the hydrophobic group, the smaller the size of the NP [31, 32]. The stability of CHPs was superior at least 2 months with no significant size and zeta potential changes, and pullulan nanoparticles can target into tumor tissue to kill cancer cell by EPR effect [33, 34].

In this study, we used pullulan (CHP) NPs hydrophobically modified with cholesterol as antitumor drug carriers to load mitoxantrone. Different sizes of mitoxantrone-loaded pullulan NPs were generated by synthesizing CHP polymers in different succinic anhydride cholesterol ester (CHS) charge ratios to study the effect of NP size on sustained release of a drug, drug release in an acidic environment, toxicity to bladder cancer cells, cell uptake efficiency, and cell migration. This experiment evaluated the size range of NPs with passive targeting to screen a suitable NP as a drug carrier and for stronger drug efficiency.

## Materials and Methods

### Reagents and Instruments

Mitoxantrone was from Aladdin Chemistry (Shanghai); the dialysis bag (BioSharp, USA, 8000~12,000 Da) was from Tianjin Junyao Biotechnology. Other reagents were from Beijing Xinze Technology.

We used the Japan F-4500 Fluorescence Spectrophotometer, J-810 circular dichroism chromatograph (Jasco Co., Japan), particle size analyzer (MALVERN, Nano 2S-90, Japan), and a projection electron microscope (JEM-100CXII, Japan).

### Synthesis and Characterization of CHP Polymer and Calculation of the Degree of Substitution of Cholesterol

The synthesis of succinic anhydride CHS was previously reported [35]. An amount of 0.5 g pullulan sample was dissolved in 15 mL dehydrated dimethyl sulfoxide for reserve. CHS (sugar unit/CHS = 0.20, 0.15, 0.05 mmol/mmol), 4-dimethylpyridine (DMAP∕CHS = 1 mmol/mmol), and 1-(3-dimethylaminop ropyl)-3-ethyl-carbodiimide hydrochloride (EDC∕CHS = 1.2 mmol/mmol) were separately dissolved in 10 mL DMSO, stirred at room temperature, and activated for 1 h; the activation reaction was dropped into the pullulan solution; and the reaction was stopped after 48 h. The reaction was dropped into 200 mL absolute ethanol, and then a white precipitate formed. Filtering was by suction, and the product was washed with appropriate amounts of ethanol, tetrahydrofuran, and diethyl ether and then dried at 80 °C. Three kinds of CHP polymers with different degrees of cholesterol substitution were obtained: CHP-1, CHP-2, and CHP-3 [36]. Pullulan polysaccharide and CHP polymer 10–20 mg were dissolved by DMSO-d6 under ultrasonic conditions, and ^1^H NMR spectra were examined. The degree of substitution of cholesterol in the CHP polymer was determined on the basis of the α-1,4 and α-1,6 glycosidic bonds and the area under the methylene peak.

### Preparation and Characterization of Drug-Loaded CHP NPs

Synthesis of mitoxantrone-loaded CHP NPs as described [37, 38], drug-loaded NPs were obtained by dialysis with 40 mg of each of the three CHP NPs substituted with various degrees of cholesterol and 4 mg mitoxantrone for backup. The newly prepared drug-loaded NPs or drug-loaded NPs dispersed in distilled water after lyophilization were dripped onto a copper grid with a carbon support film, and the filter paper was drained. Grids were placed in a desiccator, then 2% (*w*/*w*) phosphotungstic acid (2%) was added, which was negative after drying naturally, and observed by transmission electron microscopy (TEM) [38]. The solution of newly prepared drug-loaded NPs or drug-loaded NPs dispersed in distilled water after lyophilization was poured into cuvettes and placed in a particle size analyzer for detection. Each sample was processed three times to obtain an even size and even potential of NPs.

### Measurement of Drug Loading and Encapsulation Efficiency of Drug-Loaded CHP NPs

The drug loading content (LC%) and encapsulation efficiency (EE%) of mitoxantrone-loaded CHP NPs were measured as described [31, 39] as follows:

$$ \mathrm{EE}=\frac{\mathrm{The}\ \mathrm{amount}\ \mathrm{of}\ \mathrm{drug}\ \mathrm{in}\ \mathrm{the}\ \mathrm{NPs}}{\mathrm{Total}\ \mathrm{amount}\ \mathrm{of}\ \mathrm{Drug}} $$

$$ \mathrm{LC}=\frac{\mathrm{The}\ \mathrm{amount}\ \mathrm{of}\ \mathrm{Drug}\ \mathrm{in}\ \mathrm{the}\ \mathrm{NPs}}{\mathrm{The}\ \mathrm{amount}\ \mathrm{of}\ \mathrm{NPs}\ \mathrm{weight}} $$

### Study of Drug Release

The three types of mitoxantrone-loaded NPs were placed in phosphate-buffered saline (PBS) and in release media of pH = 6.8 and 4.0. Mitoxantrone release was studied in vitro by dialysis, and the accumulated release percentage (Q%) was calculated as described [40].

### Cell Lines and Culture Conditions

The murine bladder cancer cell line MB49 provided by Dr. P Guo (Institute of Urology, Xi’an Jiaotong University, Xi’an, Shaanxi, China) was cultured in DMEM (Lonza) supplemented with 10% fetal bovine serum (Hyclone, Logan, UT, USA) and 1% penicillin-streptomycin at 37 °C in humidified air containing 5% CO_2_.

### Cell Viability Assay

Cell viability was assessed by tetrazolium-based assay. Briefly, cells were seeded at 2 × 10^4^ per well in 96-well culture plates and were allowed to attach for 24 h. Different seeding densities were optimized at the beginning of the experiments. Cells were treated with different concentrations of mitoxantrone for 24 h in an incubator. Mitoxantrone at 0.0078, 0.0156, 0.03125, 0.0625, 0.125, 0.25, 0.5, and 1 μM was dissolved in DMEM supplemented with 1% fetal bovine serum. An amount of 50 μL MTT tetrazolium salt (Sigma) dissolved in Hank’s balanced solution at 2 mg/mL was added to each well with the indicated treatment and incubated in a CO_2_ incubator for 5 h. Finally, the medium was aspirated from each well and 150 μL DMSO (Sigma) was added to dissolve formazan crystals. The absorbance of each well was obtained by using a Dynatech MR5000 plate reader at test wavelength 490 nm and reference wavelength 630 nm.

IC_50_ values for mitoxantrone were determined by dose–response curves. The three concentrations of NPs (0.0625, 0.125, 0.25 μM) with three degrees of substitution were compared by MTT. The experimental procedure was the same as for mitoxantrone.

### Assessment of Apoptosis

Cell apoptotic rate was determined by flow cytometry with Annexin V-FITC/propidium iodide (PI). Briefly, treated cells were washed twice with cold PBS, then resuspended in binding buffer at 2 × 10^6^ cells/mL according to the manufacturer’s instructions. Then, 5 μL Annexin V-FITC and 5 μM PI were added into a 100-μL cell suspension and incubated for 30 min at room temperature in the dark. After adding 300 μL binding buffer, labeled cells were detected by flow cytometry within 1 h.

All early apoptotic cells (Annexin V-FITC–positive [stained green], PI-negative), necrotic cells (Annexin V-FITC–negative, PI-positive), late apoptotic cells (double positive), as well as living cells (double negative) were detected by flow cytometry (FCM) and analyzed by using Cell Quest software (Becton Dickinson). Argon laser excitation wavelength was 488 nm and emission wavelength 530 nm (FL-1 channel) for fluorescein isothiocyanate (FITC) and 670 nm (FL-3 c3 channel) for PI. Also, apoptosis was examined by fluorescence microscopy. First, 1.0 × 10^5^ cells were seeded in 96-well culture plates and after 24 h, cells were treated as above, then 24 h later, 100 μL binding buffer, 1 μL Annexin V-FITC, and 1 μLPI were added into cells at room temperature in the dark for 15 min, kept at a low temperature, and observed by fluorescence microscopy.

### Cell Migration Assay

A total of 8 × 10^5^ cells were seeded in six-well plates and allowed to reach full confluence. The monolayer was wounded by using a cocktail stick. Cells were incubated with serum-free DMEM as indicated. Digital images were taken at 0, 6, 12, 24, and 48 h. The mean area was calculated by using Image J and experiments were repeated three times.

## Results and Discussion

### CHP Conjugates and Degree of Substitution of Cholesterol

**Fig. 1:**
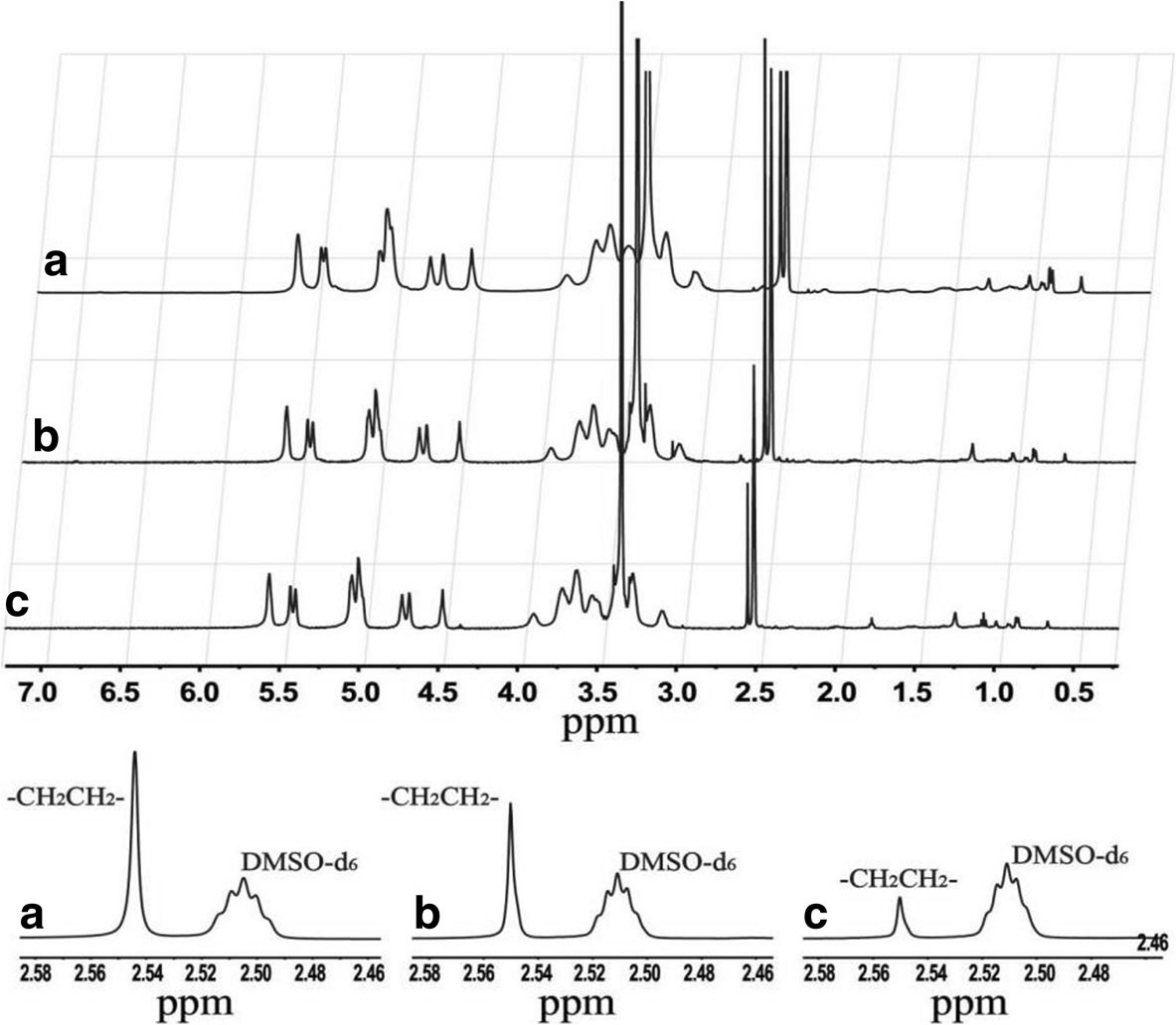
NMR spectra for CHP-1 (**a**), CHP-2 (**b**), and CHP-3 (**c**)

The ^1^H NMR value for CHP (DMSO-d6 with TMS, ppm) was 2.53 ppm (2 methylene groups, –OCH_2_CH_2_O–). Figure 1 shows ^1^H NMR spectra, confirming that cholesterol was chemically bonded to the pullulan long chain via a succinic spacer arm. The spectra for the three CHP NPs synthesized at different feed ratios (a, b, c) showed the characteristic peaks of pullulan; α-1-4 and α-1,6 glycosidic bonds were ∂_4.68_(1Hα_1–6_), ∂_5.05_(1Hα_1–4_), and ∂_2.53_ (2 methylene groups, –OCH_2_CH_2_O–), respectively, which was also easy to distinguish. New characteristic peaks appeared at 0.40 to 2.40 (hydrogen on the cholesteric skeleton), which confirmed that the three CHP polymers were successfully synthesized. The area under the peak reflects the number of atoms, and the degree of cholesteric substitution can be calculated as follows [41]:

$$ \mathrm{DS}=\frac{A_{\partial 2.53}}{4\left({A}_{\partial 4.68}+{A}_{\partial 5.05}\right)}\times 100\% $$

where the sum of A _∂4.68_ and A _∂5.05_ represents the number of sugar units, A_∂ 2.53_ is the number of hydrogen atoms in –OCH_2_CH_2_O– of the cholesteryl succinic, and A_∂ 2.53_/4 is the number of –OCH_2_CH_2_O–, that is, the number of cholesterols in succinic anhydride CHS. Thus, the above formula represents the degree of cholesteric substitution in the CHP molecule as the number of cholesteryl groups per 100 glucose units. The calculated feed ratios and molar ratios of cholesteryl and pullulan sugar units were 1/5, 3/20, and 1/20, respectively, and the degree of substitution of the three synthesized CHP-1, CHP-2, and CHP-3 polymers was 6.82%, 5.78%, and 2.74%, respectively. The degree of substitution of cholesterol on the pullulan chain increased with increasing feed ratio. However, the actual degree of substitution was lower than both feed ratios.

The pullulan chain may exist as a flexible, coiled chain in the solvent, and after the addition of a certain amount of cholesterol, the grafted cholesterol shows a larger molecular steric hindrance, which affects the further direct esterification reaction of succinyl cholesterol and the hydroxyl group on the pullulan chain. The difficulty of the reaction was significantly increased, so the degree of substitution became smaller.

### Drug-loaded CHP NPs and Their Size

**Fig. 2:**
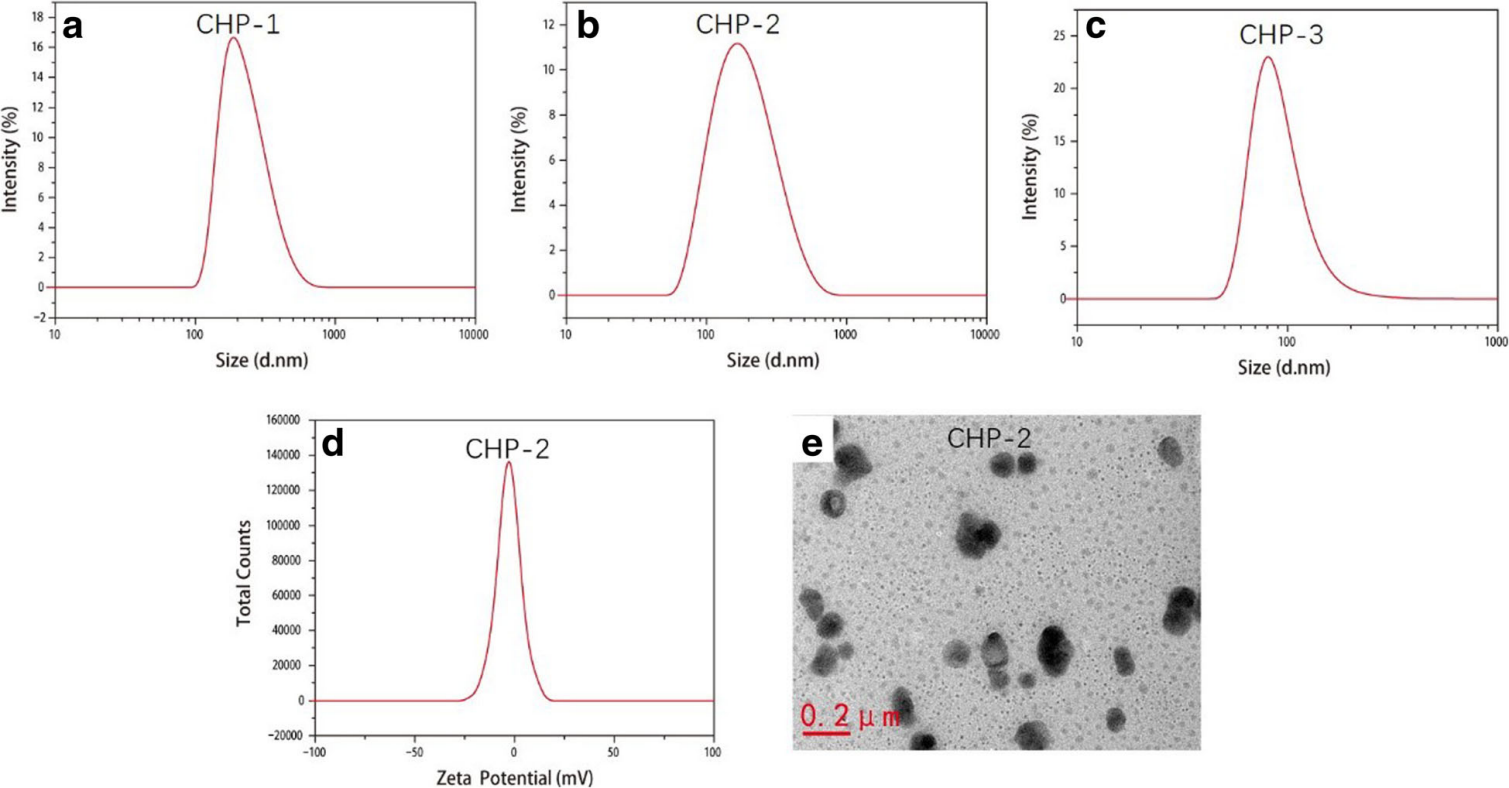
Images of NP size loaded with mitoxantrone (CHP-1 (**a**), CHP-2 (**b**), CHP-3 (**c**)), potential images (CHP-2 (**d**)), and TEM images (CHP-2 (**e**))

The sizes of the three blank CHP NPs for CHP-1, CHP-2, and CHP-3 were 79.1, 104.9, and 166.8 nm. At a certain degree of substitution, the hydrophobicity strengthened with increasing degree of substitution of cholesterol. The stronger the hydrophobicity, the better the CHP self-aggregated NPs formed a more compact hydrophobic core, which decreased the size of NPs [42]. Figure 2 shows the size of drug-loaded CHP NPs. The particle sizes for CHP-1, CHP-2, and CHP-3 were 86.4, 162.30, and 222.28 nm, respectively. In the same ratio of drugs and materials, the particle size of the drug-loaded NP with a high degree of substitution of the polymer hydrophobic group was small, but the particle diameter of the drug-loaded NP was larger than the non-encapsulated drug-containing blank NP with the same degree of substitution. As mitoxantrone enters the hydrophobic core, the particle size of the NPs is increased. In Fig. 2d, the zeta potential of the drug-loaded CHP NPs is − 1.12 mV. Fig. 2e is a TEM image showing that the drug-loaded NPs are spherical.

### Drug Release of Different-Sized Drug-Loaded NPs and Under Different Acidic Media

**Fig. 3:**
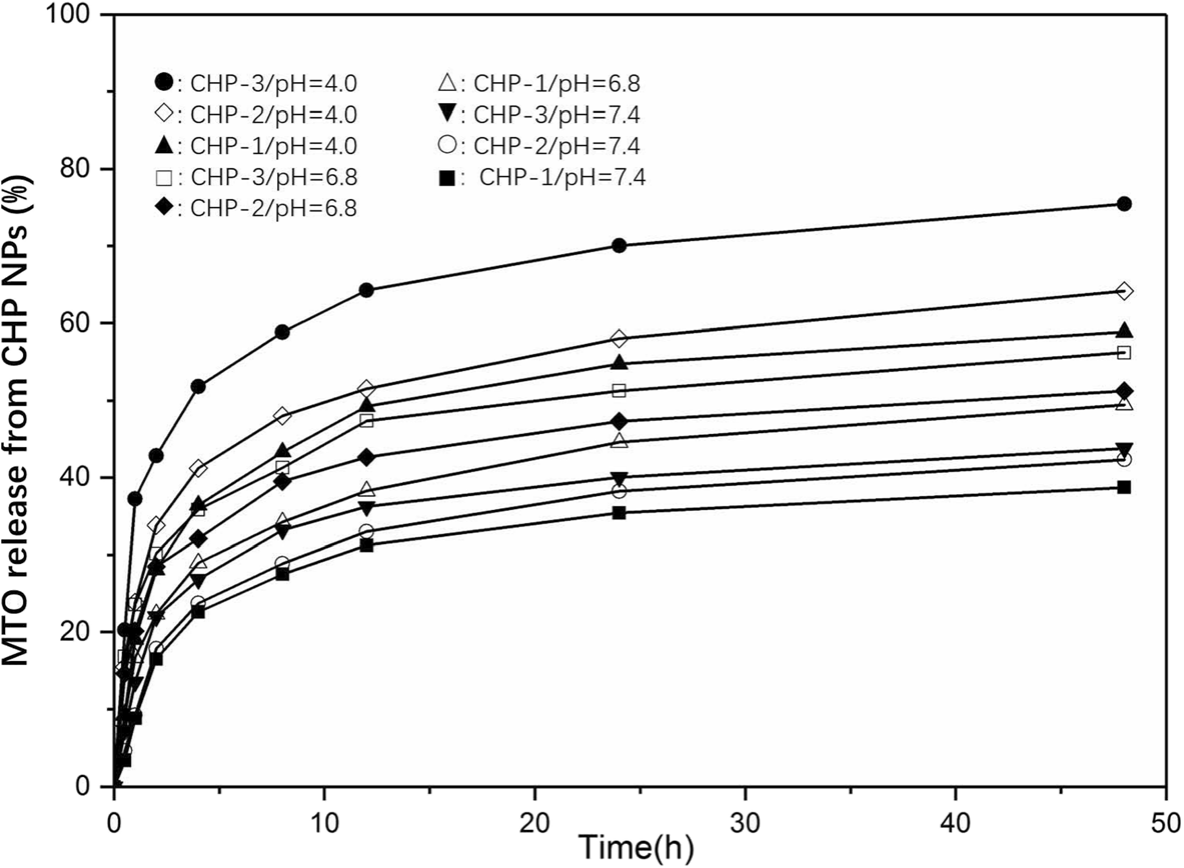
Mitoxantrone (MTO) release from pullulan NPs in phosphate buffered saline (black square: CHP-1, white circle: CHP-2, black down-pointing triangle: CHP-3), in pH 6.8 (white up-pointing triangle: CHP-1, black diamond: CHP-2, white square: CHP-3) and in pH 4.0 (black triangle: CHP-1, white diamond: CHP-2, black circle: CHP-3) at 37 °C in vitro

When the drug and CHP polymer ratios were the same, the drug loading and entrapment efficiency of the drug-loaded CHP-1, CHP-2, and CHP-3 NPs were 8.17% and 88.92%; 7.62% and 82.28%; and 4.83% and 50.67%, respectively. The higher the cholesteric hydrophobic substitution in the CHP polymer, the smaller the particle size formed and the higher the drug loading and entrapment efficiency. Figure 3 shows the drug release profiles for the three drug-loaded CHP NPs. In PBS, the drug was released for 48 h. The release rates for CHP-1, CHP-2, and CHP-3 were 38.73%, 42.35%, and 58.89%, respectively. All three NPs showed sustained release properties, but the smaller the NP size, the stronger the hydrophobicity and the slower the drug release. At pH 6.8, the drug release rates for CHP-1, CHP-2 and CHP-3 were 43.82%, 49.48%, and 64.18%, respectively. In weakly acidic conditions, CHP NPs released the drug sustainably, but the release rate significantly increased. At pH 4.0, after 48-h drug release, the drug release rates for CHP-1, CHP-2, and CHP-3 were 51.25%, 56.23%, and 75.46%, respectively. The release of the CHP NP drug was significantly faster at lower pH, especially for CHP-3 NP, the largest of the three CHP NPs.

### Cytotoxicityof Mitoxantrone-Loaded CHP NPs

**Fig. 4:**
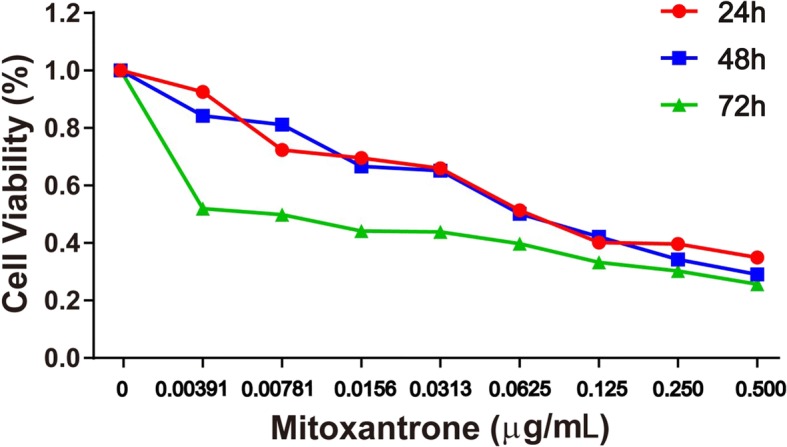
Effect of treatment with mitoxantrone and NPs on cell proliferation of bladder cancer cell line MB49. Cell viability was assessed by tetrazolium-based assay with 24-, 48-, and 72-h treatment with mitoxantrone and nano-drugs from 0 to 0.5 μg/mL on the murine bladder cancer cell line MB49

On MTT assay (Fig. 4), the IC_50_ values for mitoxantrone for inhibiting the growth of bladder cancer cells were 0.25, 0.20, and 0.06 μM at 24, 48, and 72 h, respectively (Table 1). We considered 24 h as the dosing time.

**Table 1:** 50% inhibitory concentration (IC_50_) of mitoxantrone in nano-drug preparations at various times

Times	24 h	48 h	72 h
IC_50_ (μM)	0.25	0.20	0.06

**Fig. 5:**
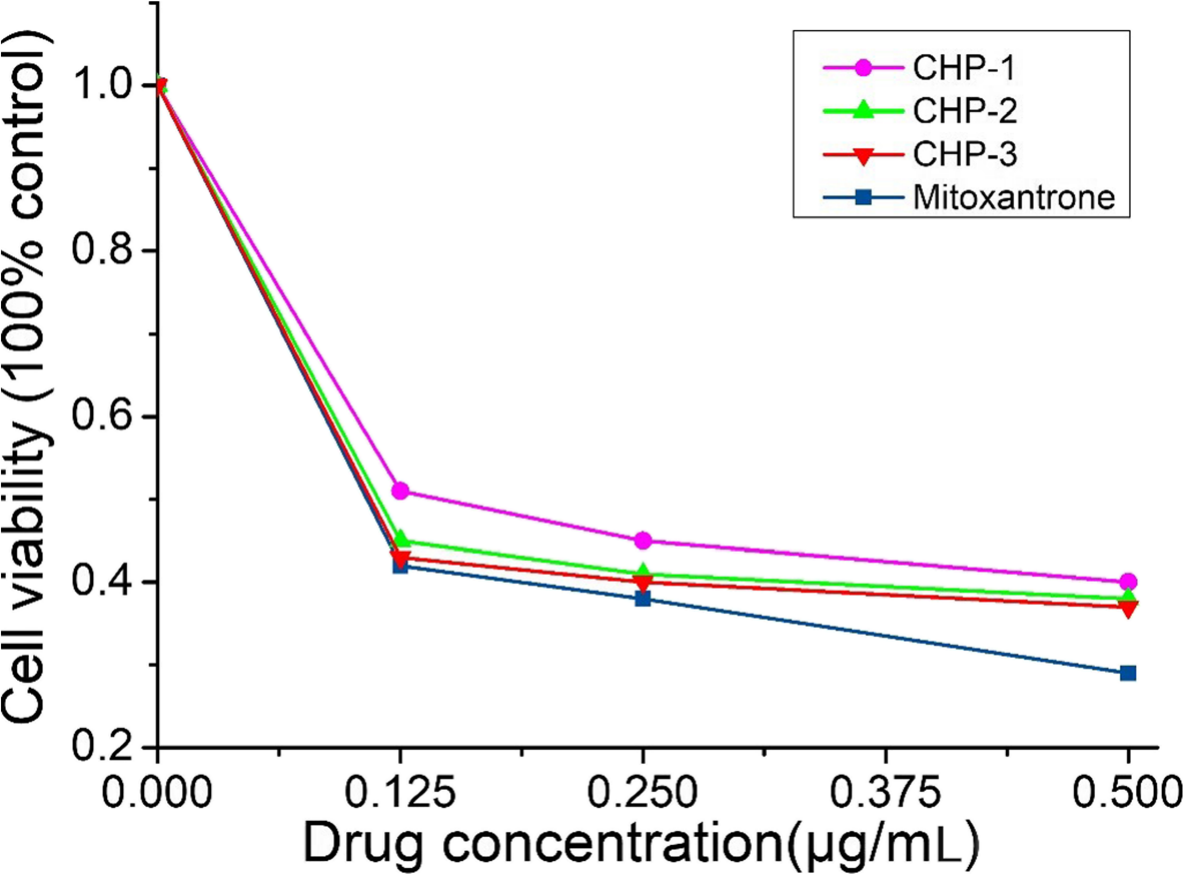
Cytotoxicity of free mitoxantrone and mitoxantrone-loaded CHP NPs at 24 h (blue square: mitoxantrone, pink circle: CHP-1, green triangle: CHP-2, red down-pointing triangle: CHP-3)

With the concentration of free mitoxantrone and mitoxantrone-CHP NPs being the same with the same administration, the results of MTT experiments in Fig. 5 show that the free mitoxantrone concentration was more toxic to bladder cancer cells than mitoxantrone-CHP NPs. In comparing the mitoxantrone-CHP NPs with the three cholesterol degrees of substitution, the most potent cytotoxic effect was CHP-3, followed by CHP-2, and the weakest was CHP-1.

Although the toxic effects of various concentrations of mitoxantrone-CHP NPs on bladder cancer cells were similar, especially CHP-2 and CHP-3, the effect of CHP-1 was significantly reduced. Each concentration of CHP-1 showed this phenomenon. Thus, the larger the mitoxantrone-CHP NP size, the stronger the cytotoxicity.

The therapeutic effect of NPs has two parts: (1) the cellular uptake of NPs and (2) NPs being released outside the cell and drugs entering the cells freely to exert their efficacy. Because free mitoxantrone has a stronger effect than mitoxantrone-CHP NPs, CHP-3 had a stronger therapeutic effect than the other two CHP NPs at the same drug dose. The release of CHP-3 was the fastest, and the therapeutic effect of CHP NPs depended mainly on the toxicity of the free mitoxantrone in cells after release of the nano-pharmaceutical preparation.

### Cell Apoptosis of Mitoxantrone-CHP NPs

**Fig. 6:**
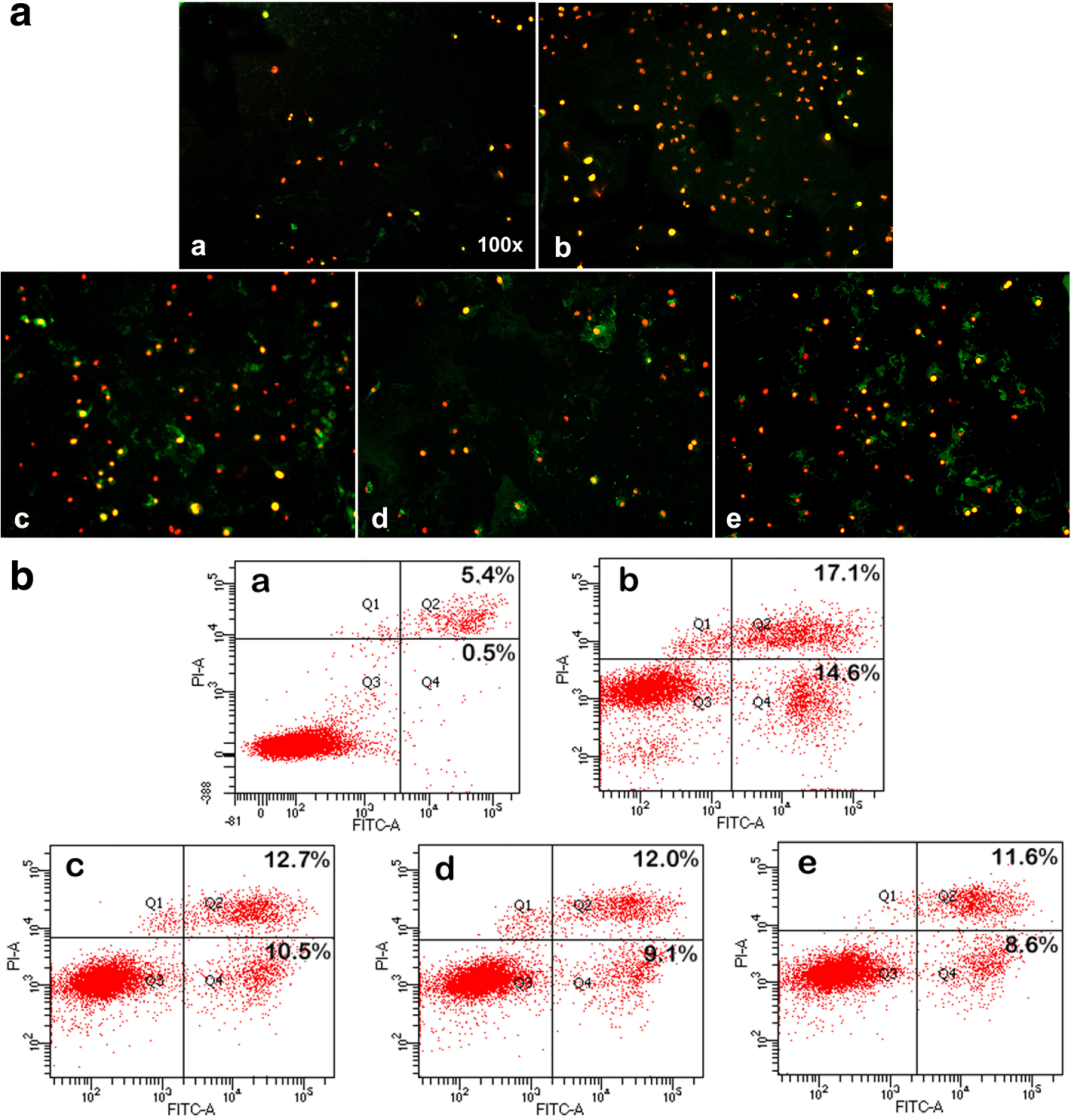
Apoptosis of mitoxantrone and nanodrugs at 24 h on MB49 bladder cancer cells(**a** DMSO, **b** mitoxantrone, **c** CHP-3, **d** CHP-2, **e** CHP-1): A. Annexin V-FITC/PI double staining was detected by fluorescence microscopy, early apoptotic cells showed Annexin V-FITC–positive staining (green), necrotic cells were PI-positive (red), and late apoptotic cells showed positive double staining (yellow). B. Apoptotic rate was determined by FCM. Live cell (Q3), early apoptotic rate (Q4), late apoptotic rate (Q2), necrotic cells (Q1).The greater the cell staining, the higher the apoptotic rate

We used immunofluorescence and flow cytometry to compare the effect of the same concentration of 0.2 μg/mL mitoxantrone and the three drug-loaded CHP NPs on apoptosis of MB49 cells. Free mitoxantrone was stronger for apoptosis than the three mitoxantrone-CHP NPs (Fig. 6). However, CHP-3 had the most potent effect, and the weakest was CHP-1. The previous MTT results were further confirmed.

### Cell Migration of Mitoxantrone-Loaded CHP NPs

**Fig. 7:**
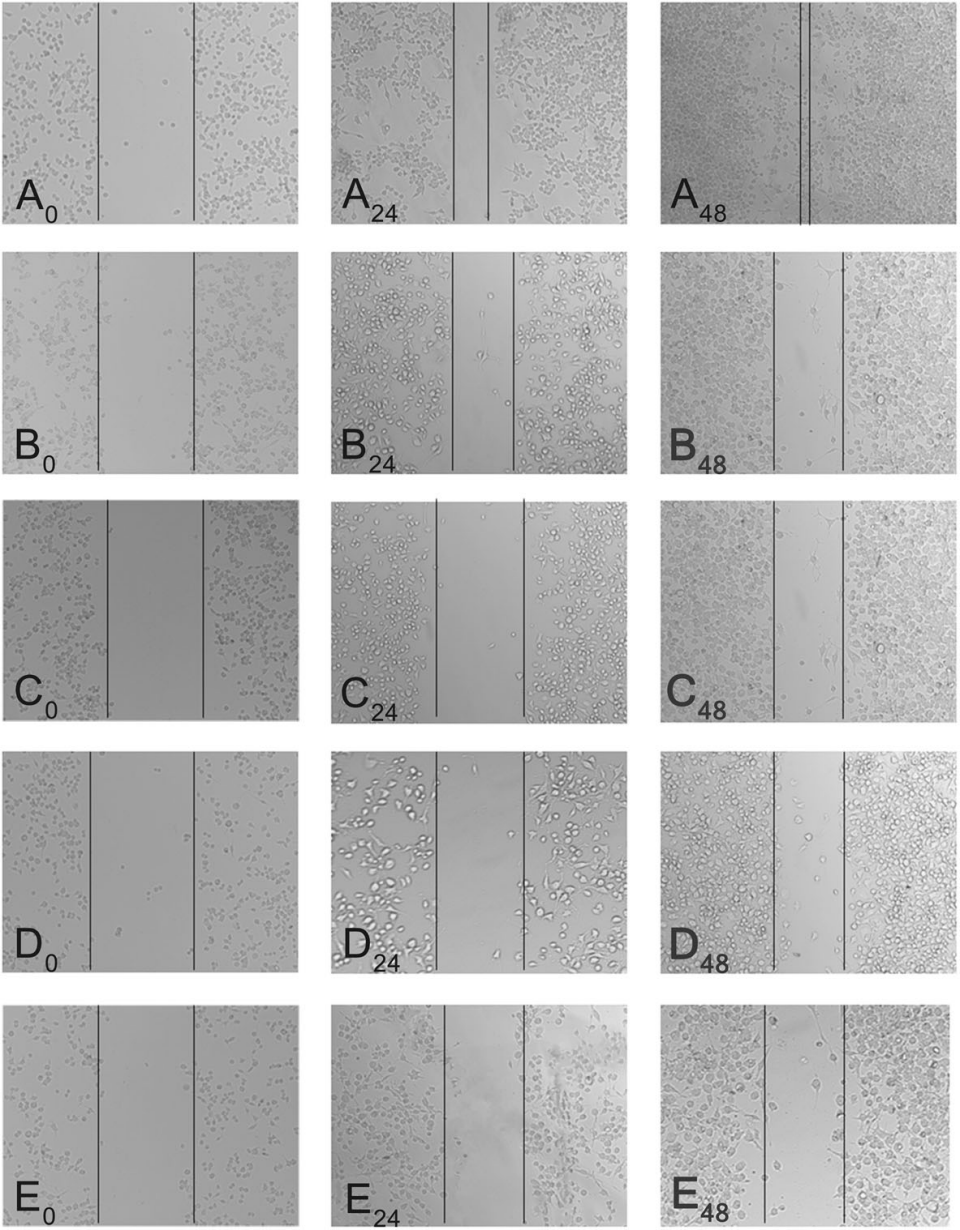
Mitoxantrone alone and mitoxantrone loaded-CHP NPs showed impaired migration on wound healing assays. **a** DMSO, **b** mitoxantrone, **c** CHP-3, **d** CHP-2, **e** CHP-1. Images showed the gap of the scratched region at different times; A_0_, A_24_, and A_48_ represent 0, 24, and 48 h of DMSO treatment, respectively

The 24- and 48-h ability of free mitoxantrone and the three CHP NPs to inhibit MB49 cell migration was observed by comparison with controls (Fig. 7). The migration inhibition was not significantly stronger for free mitoxantrone than the three CHP NPs. On MTT assay and apoptosis test, the migration inhibition was stronger for the free drug than the three CHP NPs, mainly because the free drug more easily entered cells to kill cancer cells. In the cell migration experiment also, the free drug may inhibit cell migration more efficiently than CHP nano-pharmaceuticals, which may be due to some CHP nano-pharmaceuticals not being phagocytized between cells, which results in migration resistance of cancer cells. Moreover, the three CHP NPs did not differ in inhibiting the migration of cancer cells, so the steric resistance formed by the NPs played an important role in cell migration. Therefore, drug-loaded CHPNPs inhibit cancer cells in two ways: (1) the extracellular release is the dominant way, whereby nano-drugs release the drugs outside the cells and kill cancer cells as free drugs, with CHP-3 NPs being more toxic than the other CHP NPs, and (2) CHP NPs outside cancer cells create steric resistance, therefore blocking the migration of cancer cells.

The aim of this study was to screen suitable size CHP NPs as drug carriers and to provide experimental evidence for the therapeutic action of CHP NPs. We synthesized three kinds of sterol-substituted pullulan polymers (CHPs), CHP-1, CHP-2, and CHP-3, with degree of cholesterol substitution 6.82%, 5.78%, 2.74% respectively, and diameter 86.4, 162.30, and 222.28 nm. The drug release rate of three kinds of mitoxantrone-CHP NPs for 48 h was 38.73%, 42.35%, and 58.89%, respectively. The hydrophobic substitution degree in the polymer was associated with the self-assembly process of the NPs, affecting their size and therefore drug release rate. In acid release media, the release was significantly accelerated. The larger the NP, the greater the drug release velocity. At 24 h, the IC_50_ value was 0.25 M, for the best inhibition effect of mitoxantrone on growth of bladder cancer cells. Drug-loaded CHP-3 NPs with the largest size were the most toxic to bladder cancer cells and CHP-3 NPs had the strongest effect on promoting apoptosis of the cells. All NPs could inhibit the migration of MB49 cells, but large-size CHP-3 NPs had the most powerful inhibition.

Amphiphilic polymers can self-assemble into NPs in aqueous solutions; examples are the polysaccharides pullulan and chitosan, which can be modified into amphiphilic polymers by hydrophobic modification of small molecules and self-assembled into spherical NPs in aqueous solutions with hydrophobic groups as the core and hydrophilic sugar chain shells [43, 44]. During self-assembly, hydrophobic groups are the driving force for the formation of NPs and key to the formation of their shell and core structure. The properties and molecular weight of hydrophilic groups also have an important effect on the formation and size of NPs [45, 46]. When the same polymer is modified with a small fraction of a hydrophobic group, the degree of hydrophobic substitution should be moderate, and only within a certain range can the hydrophobic substitution be self-assembled into NPs. If the degree of hydrophobic substitution is too high, the hydrophobicity of the polymer is too strong, which is not conducive to self-assembly. If the hydrophobic substitution is too low, the hydrophobic driving force is too small to form NPs [47].

**Fig. 8:**
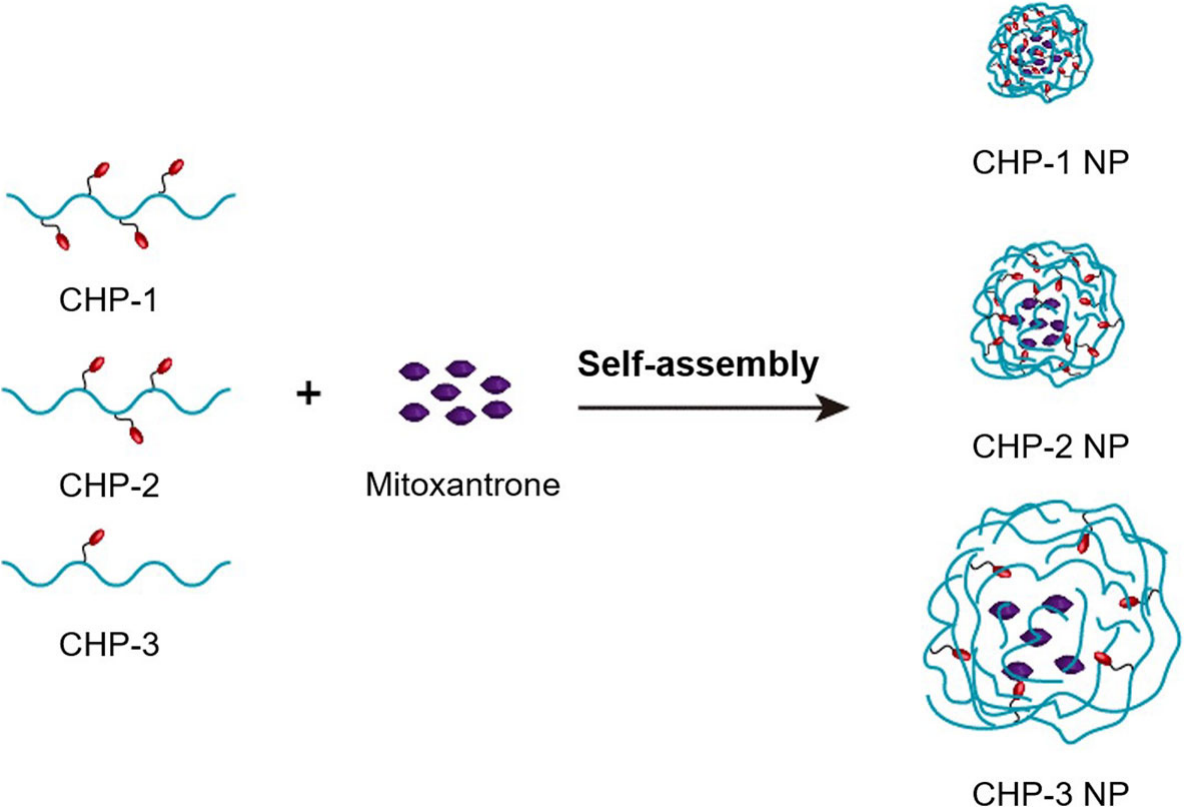
The self-assembly of mitoxantrone-loaded CHP nanoparticles (NPs)

In this study, we successfully synthesized three kinds of CHP polymers with various degrees of substitution of cholesterol by designing a suitable feed ratio, and all could self-assemble into NPs of a certain size. During self-assembly of CHP polymers, hydrophobic drugs such as mitoxantrone can be embedded in the hydrophobic center of NPs to form drug-loaded NPs (Fig. 8). The size of drug-loaded NPs is related to the degree of substitution of polymer CHP: the higher the degree of substitution, the smaller the size. The degree of substitution of polymers also affects the amount of drug loaded into NPs after self-assembly. When the ratio of polymer to drug is the same, the higher the degree of substitution, the greater the drug load [48]. Also, the ratio of polymer to drug affects the encapsulation efficiency and drug loading. Only when the feed ratio is in the proper range will the drug loading and encapsulation efficiency be relatively high [31]. Drug release of NPs directly affects their therapeutic effects, which is closely related to the kinds of nanomaterials, the surface charge and hydrophobic group of NPs, the pH value of releasing media, and the adsorption of the protein human serum albumin (HSA) in vivo [49, 50]. The drug release of mitoxantrone-loaded CHP NPs showed slow release. The drug release of CHP NPs with large size was faster and that of NPs in an acid environment was faster. The drug release rate of larger-sized NPs was more obvious and faster.

**Fig. 9:**
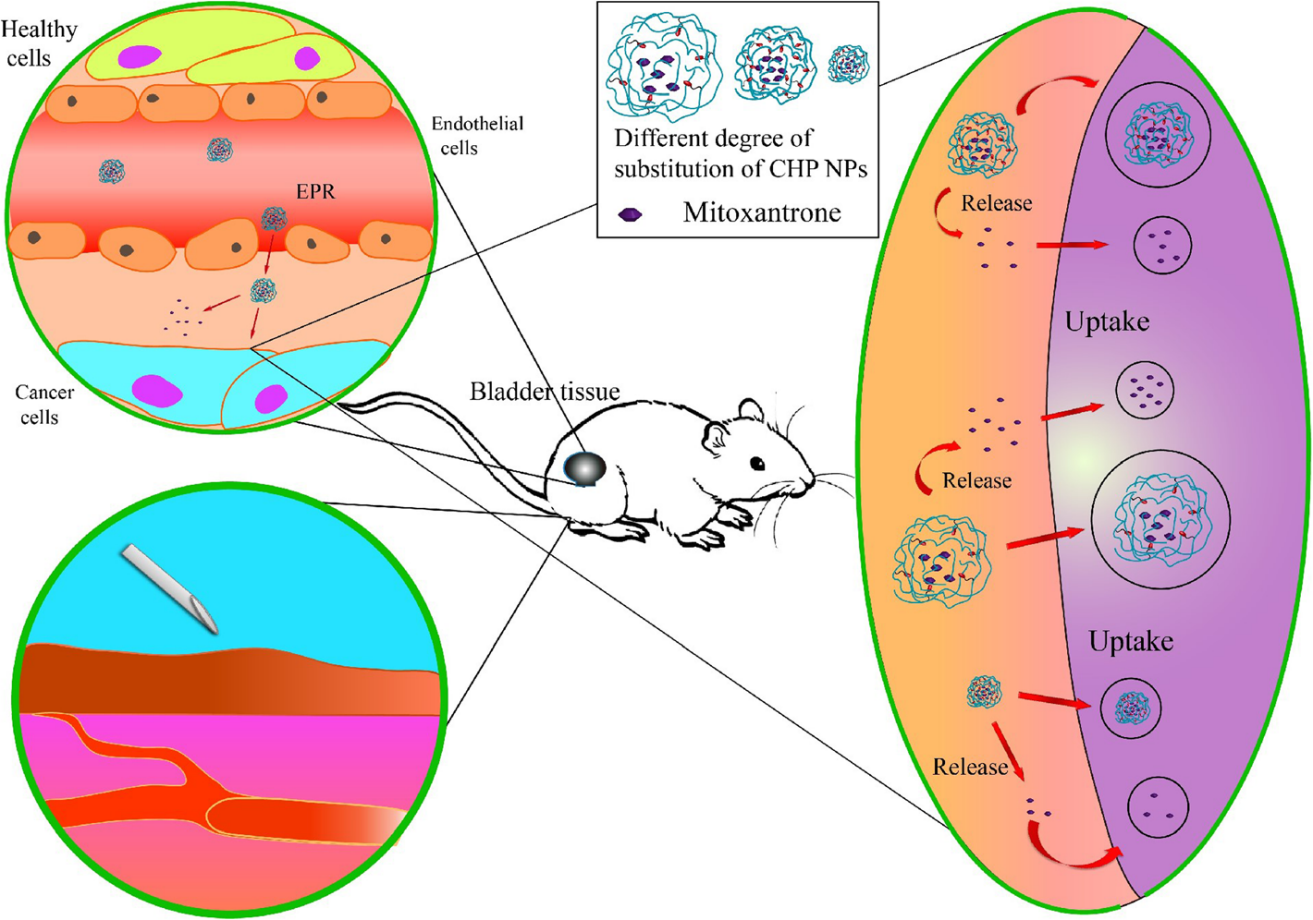
The treatment efficacy of mitoxantrone-loaded CHP NPs by mainly location release in tumor tissue

Cancer chemotherapy is the main way to treat cancer currently, but the chemotherapy drugs are not tissue-specific and are toxic to normal tissues, and some cause great damage to immune cells, which harms the overall treatment effect [51, 52]. Nanomedicine can passively target cancer tissues via the EPR effect, thereby reducing drug deposition in non-target tissues and reducing toxicity and side effects. In this study, we used bladder cancer cells as model cancer cells, and we discuss the effects of NPs and NPs size on bladder cancer. The antitumor effect was stronger for free mitoxantrone than CHP NPs; however, if the whole drug is given, mitoxantrone is not tissue-specific. The deposition and wasting of tissues and the toxicity and side effects caused by these drugs will not be as effective as nano-drug treatments. Therefore, the toxic effects on cancer cells and the inhibition of cell migration was better with the free drug than drug-loaded NPs, which does not indicate that the overall therapeutic effect of CHP nanometers is not as good as that of free mitoxantrone. We point out the effect of hydrophobic degree of substitution on the size of nanoscale drugs and the effect of nano size on drug loading, drug release, cytotoxicity and cancer cell migration. After the NPs are passively targeted to cancer tissue via the EPR effect, the therapeutic effectiveness of drug-loaded NPs is mainly derived from the release of drugs in the tissue and the release of NPs to cells (Fig. 9). The therapeutic effect of CHP NPs is whether its extracellular or intracellular release plays the dominant role. From the cell experiments, the size of CHP NPs has a strong effect: with large size, drugs are released more, but the amount of the drug is the same. Therefore, the therapeutic effect of CHP-NPs may depend mainly on the release in the tissue instead of cell uptake.

Many classical NPs are used as drug carriers, and the CHP NPs we prepared are superior to others. For example, biogenetic NPs (such as exosome, extracellular vesicles-mimetic, modularized extracellular vesicles) are difficult to prepare [53]. The target distribution of common liposomes is not ideal and its instability is still a problem [54]. Inorganic NPs such as quantum dot NPs are very stable, but as foreign matter, their biocompatibility is poor, which may cause side effects to humans [55]. CHP NPs are easy to prepare and we can control their size by controlling the degree of hydrophobic substitution [48]. Because they can be directly degraded by amylase in vivo, they have good biocompatibility [56]. In addition, CHP NPs have good stability and excellent drug release properties [57].The disadvantage is that they will inevitably be swallowed in part by the mononuclear phagocytic system [58]. More research is needed to reduce the removal by the system and improve the effective blood concentration of NPs.

## Conclusion

The size of mitoxantrone-loaded CHP NPs is related to the degree of cholesterol substitution in the polymer. The higher the hydrophobicity substitution degree, the smaller the size, and the greater the drug loading and encapsulation efficiency, and the slower the drug release. Under acidic conditions, the stronger the acidity, the faster the release of CHP NPs. Moreover, the release of NPs with larger size is best and larger-sized NPs can inhibit the growth of bladder cells and their migration better than smaller-sized NPs. CHP NPs kill cancer cells mainly by the release of nanoscale drugs outside the cell.
